# Structurally distinct external solvent-exposed domains drive replication of major human prions

**DOI:** 10.1371/journal.ppat.1009642

**Published:** 2021-06-17

**Authors:** Mohammad Khursheed Siddiqi, Chae Kim, Tracy Haldiman, Miroslava Kacirova, Benlian Wang, Jen Bohon, Mark R. Chance, Janna Kiselar, Jiri G. Safar

**Affiliations:** 1 Department of Pathology, Case Western Reserve University, Cleveland, Ohio, United States of America; 2 Department of Nutrition, Case Western Reserve University, Cleveland, Ohio, United States of America; 3 Center for Proteomics and Bioinformatics, Case Center for Synchrotron Biosciences, Brookhaven National Laboratory, Upton, New York, United States of America; 4 Department of Neurology, Case Western Reserve University, Cleveland, Ohio, United States of America; Istituto Superiore di Sanità, ITALY

## Abstract

There is a limited understanding of structural attributes that encode the iatrogenic transmissibility and various phenotypes of prions causing the most common human prion disease, sporadic Creutzfeldt-Jakob disease (sCJD). Here we report the detailed structural differences between major sCJD MM1, MM2, and VV2 prions determined with two complementary synchrotron hydroxyl radical footprinting techniques—mass spectrometry (MS) and conformation dependent immunoassay (CDI) with a panel of Europium-labeled antibodies. Both approaches clearly demonstrate that the phenotypically distant prions differ in a major way with regard to their structural organization, and synchrotron-generated hydroxyl radicals progressively inhibit their seeding potency in a strain and structure-specific manner. Moreover, the seeding rate of sCJD prions is primarily determined by strain-specific structural organization of solvent-exposed external domains of human prion particles that control the seeding activity. Structural characteristics of human prion strains suggest that subtle changes in the organization of surface domains play a critical role as a determinant of human prion infectivity, propagation rate, and targeting of specific brain structures.

## Author summary

Sporadic human prion diseases are conceivably the most heterogenous neurodegenerative disorders and a growing body of research indicates that they are caused by distinct strains of prions. By parallel monitoring their replication potency and progressive hydroxyl radical modification of amino acid side chains during synchrotron irradiation, we identified major differences in the structural organization that correlate with distinct inactivation susceptibility of a given human prion strain. Furthermore, our data demonstrated, for the first time, that seeding activity of different strains of infectious brain-derived human prions is primarily function of distinct solvent-exposed structural domains, and implicate them in the initial binding of cellular isoform of prion protein (PrPC) as a critical step in human prion replication and infectivity.

## Introduction

Prions are a distinct class of infectious agents that are composed of self-replicating misfolded protein aggregates and are causing invariably fatal neurodegenerative diseases affecting both humans and animals. The rapidly progressing neurodegeneration is caused by a pathogenic protein, designated PrP^Sc^ [1], a misfolded isoform of the normal cellular prion protein [2–7], PrP^C^. Humans prion diseases include Gerstmann-Sträussler-Scheinker disease (GSS), fatal insomnia (FI), kuru, variably protease-sensitive prionopathy (VPSPR), and Creutzfeldt-Jakob disease (CJD) [8–11]. The sporadic form of Creutzfeldt-Jakob disease (sCJD) is the most prevalent and accounts for ~90% of cases globally [9–11]. In contrast to stable cloned laboratory and animal prions, human prions are perhaps the most phenotypically diverse; they exist in a broad spectrum of strains causing at least eight diverse clinicopathological disease phenotypes, and may undergo further mutation and evolution by a conformational selection mechanism [12]. Codon 129 (M or V) polymorphism in *PRNP* gene has a major impact on disease phenotype and progression rate and is used together with 21kDa (type 1) or 19kDa (type 2) mass of protease resistant core of sCJD prions into clinicopathological classification, with sCJD MM1, MM2, and VV2 which are representing ~80% of all cases [9–11].

A number of studies have demonstrated that rodent prions can be generated in vitro from bacterially-expressed recombinant mouse or Syrian hamster PrP [13–17], and these experiments played a fundamentally important role in providing the ultimate proof for the protein-only hypothesis of prion diseases [15, 16, 18–21]. However, our early data using hydrogen/deuterium exchange and mass spectrometry indicated that human prions causing sporadic Creutzfeldt-Jakob disease (sCJD) differ in a major way from both cloned laboratory prions and synthetic prion amyloids, and phenotypically distant MM1 sCJD prions further differ in their structural organization from MM2 sCJD prions [22, 23]. Additionally, by comparing the structural organization of human prions that we recently synthesized in vitro [24] with that of parent sCJD MM1 prions and noninfectious human prion protein amyloid, we have identified critical differences in PrP that are important for the initiation of replication *in vivo*, i.e. their infectivity. Moreover, these data indicated that, in contrast to previous observations on yeast and some murine prion strains, the replication rate of sCJD prions is primarily determined not by conformational stability, but by their unique structural features that control the growth rate of prion protein aggregates [22]. Although these structural and functional insights implicate the initial interaction between prion seed and PrP^C^ substrate as a critical step in human prion replication and propagation, the structural elements (domains) driving this process, and the role of different codon 129M or 129V polymorphisms are not known. A growing body of evidence indicates that the improved understanding of this mechanism in human CJD prions will have major implications for other age-related neurodegenerative diseases linked to other misfolded proteins, including an ongoing debate about their potential transmissibility [25–27].

In order to gain insight into the impact of the structural organization of major human brain derived sCJD MM1, MM2, and VV2 prion strains on their replication and inactivation, we employed two different synchrotron hydroxyl radical (^*•*^OH) footprinting techniques. The first is a novel epitope interference study, where the affinity of a panel of monoclonal antibodies with linear epitopes in PrP monomers and sCJD prions is progressively altered by modifying key amino acids side chains by hydroxyl radicals produced with increasing doses of synchrotron radiation, and the changes are monitored by quantitative and conformationally dependent immunoassays (CDIs). The differences in kinetics of epitope modification evaluated across both PrP monomer forms and sCJD prions indicate differences in accessibility to solvent due to the differences in conformation. Further, the hydroxyl radical induced modifications are assessed after proteolysis with mass spectrometry (MS) that provide the data on PrP monomers and sCJD MM1, MM2 and VV2 prions at a single residue resolution. Both footprinting techniques indicate that the distribution of critical surface amino acid residues is an important determinant of the structural heterogeneity in human brain derived sCJD MM1, MM2 and VV2 strains and is responsible for differences in replication rate, resistance to inactivation, and pathological targeting of different brain structures observed in sCJD patients.

## Materials and methods

### Ethics statement

All procedures were strictly implemented under the standard protocols (IRB Number 03-14-28) approved by the Institutional Review Board at Schools of Medicine, Case Western Reserve University, and University Hospitals Case Medical Centre. For all the cases associated with our investigation, written informed consents for research were acquired from patients’ or legal guardians, and the material during research had appropriate ethical approval for use in this project. All patients’ data and samples were coded, anonymized, and operated in accordance with NIH guidelines in order to protect patients’ identities.

### Sporadic CJD (sCJD) case demographics

**Fig 1:**
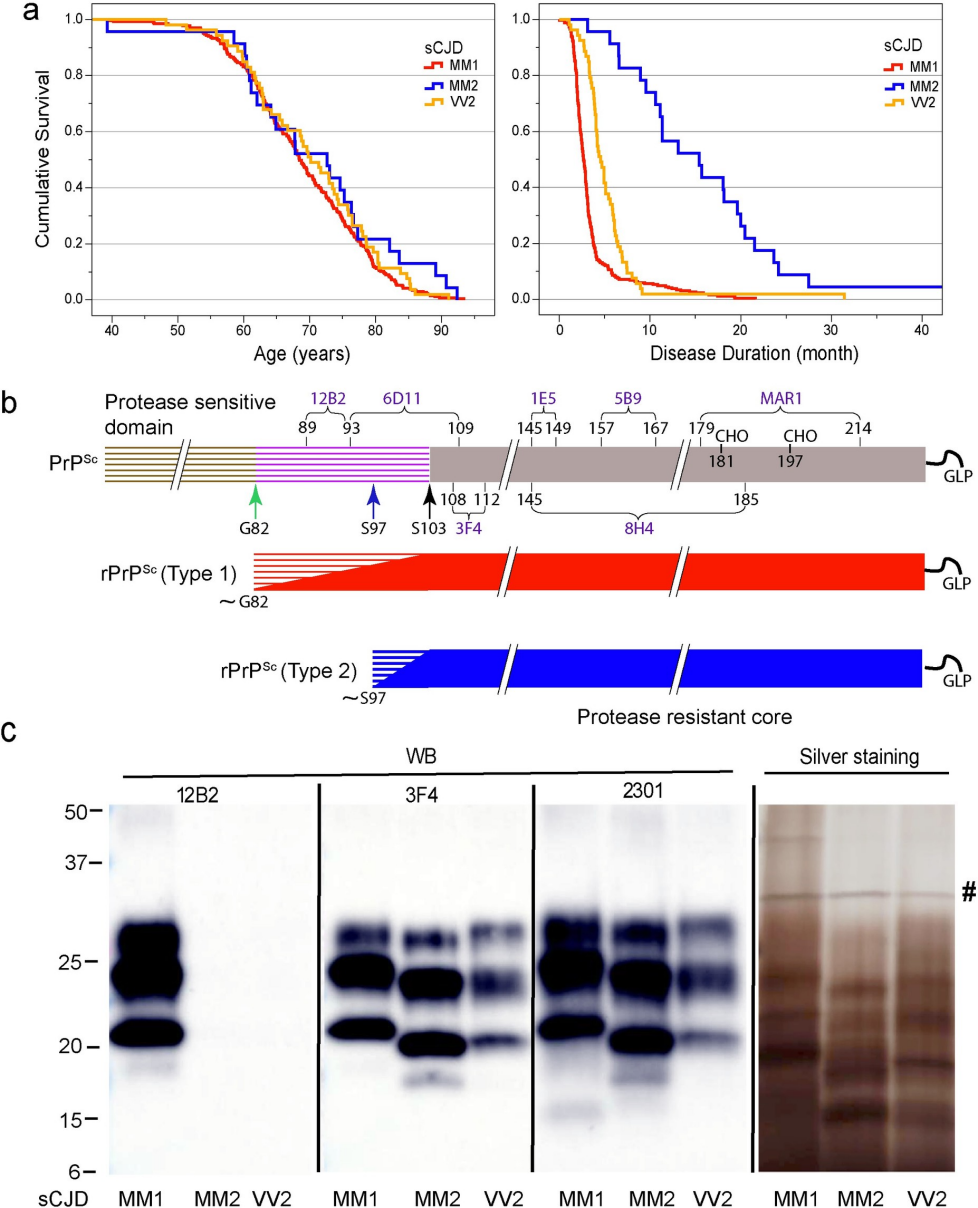
Distinct disease duration, schematic representation, and molecular characteristics of sCJD MM1, MM2, and VV2 prions. (**a**) The age (left panel) and disease duration (right panel) in 343 cases of sporadic CJD MM1, MM2, and VV2 prion disease; (**b**) Outline of classification of Type 1 and Type 2 human prions based on proteolytic fragmentation of PrP^Sc^ [75, 89]. Major cleavage sites by PK are indicated by arrows; GLP–glycolipid; CHO- complex N-glycosylation chains. The codes above light blue brackets represent monoclonal antibodies used in differentiation of various domains of human prions, and the numbers below these brackets indicate linear epitopes recognized by these antibodies. (**c**) Distinct glycosylation and Proteinase K fragmentation patterns of purified human MM1, MM2, and VV2 sCJD rPrP^Sc^ (homozygous for methionine [M] or valine [V] in codon 129) used in structural studies. To differentiate Type 1, Type 2 prions, and their C-terminal fragments, Western blots of purified rPrP^Sc^ (fraction 8; F8) of MM1, MM2, and VV2 sCJD rPrPSc were developed with mAb 12B2 (epitope residues 89–93) [90], mAb 3F4 (epitope residues 108–112) [47], and rabbit polyclonal antibody 2301 (epitope residues 220–231) [91]. (**c**) Distinct fragmentation patterns of purified MM1, MM2, and VV2 sCJD prions in silver stained SDS-PAGE. The symbol (**#**) indicates bands corresponding to PK. The molecular weights of marker proteins are in kDa.

From the collection of samples obtained from 343 patients with an unequivocal neuropathological diagnosis of sporadic CJD MM1, CJD MM2, and CJD VV2, we selected one case that is representative of each neuropathology group (**Fig 1**) and displayed ≥99% pure Type 1 or Type 2 proteinase K-resistant PrP^Sc^ (rPrP^Sc^), as detected by both conformation dependent immunoassay (CDI) and Western blots [11, 28–30]. The disease duration in these representative cases, as well as biochemical characteristics of brain PrP^Sc^ associated with them (levels of total PrP^Sc^ and rPrP^Sc^, size of PrP^Sc^ particles, and conformational stability of PrP^Sc^) correspond to the respective median values reported previously for each group [29, 30] (**Table 1**).

**Table 1:** Demographics, molecular characteristics, and synchrotron hydroxyl radical inactivation of different strains of sCJD prions. Conformational stability assay (CSA) of prions is expressed in a midpoint concentration of the GdnHCl in the transition from folded to unfolded state [24, 29, 30, 39].

Parameter	Unit	sCJD MM1	sCJD MM2	sCJD VV2
**Age**	years	57	73	63
**Sex**	F/M	F	F	M
**Disease Duration**	month	2	46	6
**Postmortem Interval**	hrs	2	72	24
***PRNP codon 129***	M/V	MM	MM	VV
**Neuropathology Classification**	-	sCJDMM(MV)1	sCJDMM2C	sCJDVV2
**Mass of Unglycosylated rPrP**^**Sc**^	kDa	21	19	19
**CSA GdnHCl**_**1/2**_	[M]	2.95±0.09	1.64±0.06	2.76±0.15
**Synchrotron Irradiation Time 0ms**	Log_10_ SD_50_/μg	8.29	7.84	8.04
**30ms**		7.84	6.84	7.84
**90ms**		7.34	6.64	7.34
**200ms**		6.39	5.89	6.39
**Prion Irradiation half-life**	ms	19.4	7.6	43.2

### Purification of MM1, MM2, and VV2 prions

The purification of PrP^Sc^ from human brains was adopted for human prions [22] from previously described purification of 263K prions from Syrian hamster brains [31–33]. Briefly, CJD brain cortex slices weighing ~24g were disrupted to final 5% (w/v) homogenate in ice-cold PBS (pH 7.4) containing 2% (w/v) Sarkosyl by three 50-s cycles in Omni TH-01 homogenizer equipped with disposable plastic probes (Thermo Fisher Scientific, Waltham, Massachusetts) and clarified for 5min at 500xg in Allegra centrifuge (Beckman Coulter, Brea, California) equipped with S-2415 rotor. The clarified 5% brain homogenate was spun for 30min at 185,000xg at 4°C in Optima XPN-100 ultracentrifuge equipped with Ti 50.2 rotor (Beckman Coulter, Brea, California), and the pellets were resuspended in PBS, pH 7.4 containing 10% (w/v) NaCl and 1% Sarkosyl. After incubation in Eppendorf Thermomixer (Eppendorf, Hauppauge, NY) for 2 h at 37°C with 600rpm shaking, the samples were spun for 2hrs at 26,000xg and 4°C in F2402H rotor of Beckman Allegra centrifuge. The partially purified samples containing ~10μg of human PrP^Sc^ were resuspended in PBS, pH7.4 containing 2mM CaCl_2_ and 2% Sarkosyl, sonicated in a sonication bath (3x5s), and incubated for 4hrs at 37°C with 70μg/ml of Collagenase (Worthington Biochemical Corporation) and shaking at 600rpm in Eppendorf Thermomixer. After adding MgCl_2_ to a final 5mM concentration, the samples were incubated with 50IU/ml of Benzonase (Novagen/EMP) for additional 1hr at 37°C, followed by 1hr incubation with Proteinase K (Amresco, Solon, OH/ Invitrogen) at 37°C and 1:50 enzyme/total protein ratio (w/w). The Proteinase K was blocked with protease inhibitor (PI) cocktail containing 0.5mM PMSF, and 2μg/ml of aprotinin and leupeptin, respectively. The pellet obtained after centrifugation (30min, 26,000xg, 4°C) in Allegra centrifuge equipped with F2402H rotor was resuspended in PBS containing 2% Sarkosyl and PI cocktail (1:1000, v/v), and delipidated overnight with four volumes of Methanol/Chloroform (2:1, v/v) at -20°C. Finally, the sample was collected by centrifugation, resuspended in water containing 0.1% Sarkosyl and stored at -80°C for further analysis.

### Synchrotron radiolysis

Radiolysis experiments were performed at the 17-BM X-ray footprinting (XFP) beamline of the National Synchrotron Light Source II, at Brookhaven National Laboratory (NY, USA) and the approved biosafety protocols covering the human prion experiments are available upon request. The irradiation time in X-ray beam with 5–16 keV energy, current 300 mA, and no Aluminum shielding was optimized by using Alexa-488 fluorophore assay [34]. The 1μg aliquots of rPrP^C^ (129M and 129V) and sCJD brain-derived rPrP^Sc^ (MM1, MM2 and VV2) in closed thin-wall PCR (Axygen) tubes were exposed for 0–70 ms and 0–200 ms, respectively. After irradiation, samples were immediately quenched with methionine amide (20mM, final concentration) to prevent any secondary oxidation by left over radicals [35]; and stored at -80°C until they were studied.

### Molecular characteristics and physicochemical properties of purified sCJD prions

The purified rPrP^Sc^ was analyzed by SDS PAGE followed by silver staining and western blots, and by conformation-stability assay (CSA). The latter assay was performed as described previously [30, 36] with the following minor modifications. First, we used white Lumitrac 600 High Binding Plates (E&K Scientific, Santa Clara, California) coated with mAb 8H4 (epitope 145–185)[37] in 200 mM NaH_2_PO_4_ containing 0.03% (w/v) NaN_3_, pH 7.5. Second, aliquots of 20 μl from each fraction containing 0.007% (v/v) of Patent Blue V (Sigma) were directly loaded into wells of white strip plates prefilled with 200μl of Assay Buffer (Perkin Elmer, Waltham, Massachusetts). Finally, the captured PrP was detected by a europium-conjugated [38, 39] anti-PrP mAb 3F4 (epitope 108–112) and the time-resolved fluorescence (TRF) signals were measured by the multi-mode microplate reader PHERAstar Plus (BMG LabTech, Durham, North Carolina). The recHuPrP(90–231,129M) and PrP(23–231,129V) used as a calibrant in the CDI was prepared and purified as described previously [40]. The conformational stability of rPrP^Sc^ was determined as described previously [30, 39] and the raw CDI signal was converted into the apparent fractional change and fitted by least square method with a sigmoidal transition model to determine GdnHCl concentration where 50% of PrP^Sc^ is unfolded ([Gdn HCl]_1/2_) [30].

### Seeding activity of human prions in second generation real-time quaking-induced conversion (RT-QuIC)

The second generation RT-QuIC for human prions, purification, and refolding of prion protein substrate were performed as previously described [41] with minor modifications. Briefly, 98μL of fresh RT-QuIC reaction mix (10 mM phosphate buffer pH 7.4; 300 mM NaCl; 0.1 mg/mL recombinant PrP; 10 μM Thioflavin T and 1 mM EDTA) was loaded into the wells of a black 96-well clear bottom plate (Nunc). The wells were then seeded with 2 μL of the serially diluted brain homogenate (BH) control or synchrotron irradiated purified prions for a final volume of 100 μL. All samples contained 0.1% SDS resulting in final concentration of 0.002% final concentration of SDS in the rection mix. Plates were sealed (Nalgene Nunc International sealer) and incubated in a BMG FLUOstar Omega plate reader (BMG Labtech, Ortenberg, Germany) at 55°C for the designated period of 60hrs with cycles of 1 min shaking (700rpm double orbital) and 1 min rest through the incubation. ThT fluorescence measurements (450±10nm excitation and 480±10nm emission; bottom read) were taken every 45 minutes. The ThT positive wells were determined as described for prion diagnostic applications [41], with wells concluded to be positive if the ThT fluorescence exceeded the cutoff calculated as a mean ± 4* standard deviations (SD) of all prion-negative wells on a given plate.

### Seeding Activity (SD_50_) calculations

The Spearman-Kärber analysis analogous to calculations of 50% prion lethal dose (LD_50_) in bioassays [36, 42–45] was used to estimate a seeding dose or dilution at which 50% of the wells became ThT positive (SD_50_) [46]. Each sample corresponding to the 0, 30, 90, and 200ms of synchrotron irradiation was 5-fold serially diluted and each dilution was tested in four wells in RT QuIC. The 50% seeding end-point was calculated using the following formula: x_p_  =   1+1/2d - d∑p (where x_p_   =   1 is the highest log dilution giving all positive responses; d   =   log dilution factor; p   =   proportion positive at a given dose; ∑p  =   the sum of values of p for x_p_   =   1 and all higher dilutions.

### Synchrotron •OH epitope modification monitored with direct Conformation Dependent Immunoassay (CDI)

**Fig 2:**
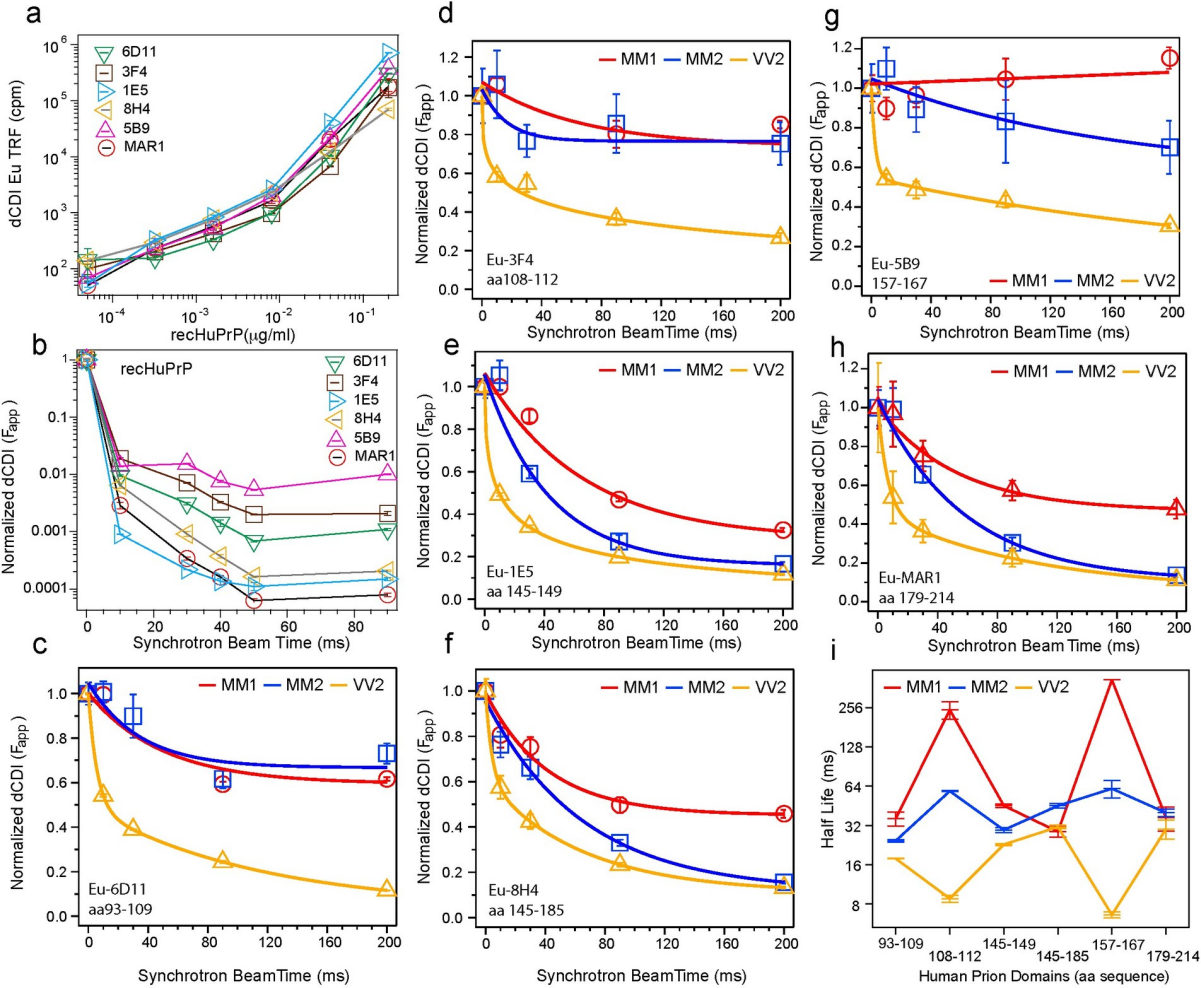
The ^*•*^OH modification rates of different antibody epitopes in recombinant human monomeric α-helical PrP (recHuPrP) and in MM1, MM2, and VV2 sCJD prions monitored with dCDI. (**a**) Calibration of dCDI with non-irradiated recHuPrP and Europium-labeled mAb 6D11 (aa 93–109), 3F4 (aa 108–112), 1E5 (aa 145–149), 8H4 (aa 145–185), 5B9 (aa 157–167) and MAR1 (aa 179–214). (**b**) Parallel rapid drop in normalized antibody dCDI signals of synchrotron irradiated recHuPrP. Time course of epitope modification monitored in sCJD MM1 (red), MM2 (blue) and VV2 (yellow) prions with mAb (**c**) Eu-6D11, (**d**) Eu-3F4, (**e**)Eu-1E5, (**f**) Eu-8H4, (**g**) Eu-5B9, and (**h**) Eu-MAR1. (**i**) Half-life of different epitopes in MM1 (red), MM2 (blue) and VV2 (yellow) sCJD prions in synchrotron beam. Each dCDI datapoint is an average ± standard error obtained in triplicate experiments normalized from the time resolved fluorescence (TRF) signal of europium in counts per minute (cpm).

The direct CDI format for human prions was performed as described previously [23, 24, 29, 30, 36] with the following minor modifications. All antibodies were Europium-labeled according to our previously published protocol [39] resulting in a density of 4.3–6.3 Eu per IgG, and calibrated against recombinant human prion protein and diluted to obtain the same signal (**Fig 2A**). Synchrotron irradiated monomeric recombinant PrP and brain derived sCJD MM1, MM2, VV2 prions were diluted in 4M GdnHCl to a constant concentration and then heated at 80°C for 5min. Denatured samples were further diluted in PBS to a final 0.2M GdnHCl and loaded onto a 96-well high-binding polystyrene plate. Sample loaded plates were incubated overnight at 4°C and blocked with either casein (Surmodics, Eden Prairie, MN) solution (1x casein, 0.05% Tween 20 and 6% sorbitol) or BSA (0.25% BSA, 0.05% Tween 20 and 6% sorbitol) in PBS. The blocked plates were incubated for two hours with Europium conjugated antibodies: Eu-3F4[47](Biolegend, San Diego, CA), epitope aa108-112; Eu-6D11, (Biolegend, San Diego, CA), epitope aa93-109; Eu-8H4[37], epitope aa145-185, (gift from Dr. Man-Sun Sy, CWRU); Eu-1E5, (Santa Cruz Biotechnology, Dallas, TX), epitope aa145-149; Eu-5B9, (Dianova Gmbh, Hamburg, GE), epitope aa157-167; and Eu-MAR1[48], epitope aa179-214 (CSL Behring BMGH), and detected by monitoring the time-resolved fluorescence signal using multi-mode microplate reader PHERAstar Plus (BMG Lab Tech, Durham, NC).

The modification rate constant for each specific epitope was calculated by fitting the fraction unmodified for this modification site to the equation: *F*_u_(*t*) = *F*_u_(0)e^-*kt*^, where *F*_u_(0) and *F*_u_(*t*) are the fraction of unmodified epitope at time zero and time t, respectively; and *k* is first order rate constant [49, 50]. The half-life (*t*_1/2_) for modification of amino acid residues in a given antibody epitope was calculated using equation: *t*_1/2_ (sec) = ln2/*k*, where *k* (sec^-1^) is the rate constant [51, 52].

### Mass spectrometry (MS) analysis of ^*•*^OH footprinting

Before proteolysis, all samples were denatured and reduced by the addition of 96% formic acid (FA) and 4mM tris 2-carboxyethylphosphine (TCEP). The supernatant, dried overnight in a vacuum desiccator filled with solid Na_2_CO_3_, was solubilized in 4.5% (v/v) FA and 4mM TCEP, sonicated, mixed with pepsin (Promega) at a pepsin-to-protein ratio (w/w) of 1:5, and incubated for 5hrs at 37°C with continuous shaking at 350rpm. Pepsin was inactivated by heating the sample at 95°C for 3 min.

Identification and quantification of oxidative sites were performed by LC-MS analysis using an Orbitrap Eclipse mass spectrometer (Thermo Scientific, CA) interfaced with a Waters nanoAcquity UPLC system (Waters, MA). Proteolytic peptides (~600 ng in 8μl; ~300ng in 4μl) were loaded on a trap column (180 μm × 20 mm packed with C18 Symmetry, 5 μm, 100 Å; Waters, MA) to desalt and concentrate peptides. Peptide mixture was separated on a reverse phase column (75 μm x 250 mm column packed with C18 BEH130, 1.7 μm, 130 Å; Waters, MA) using a linear gradient of 0 to 32% mobile phase B (0.1% formic acid and acetonitrile) vs. mobile phase A (100% water/0.1% formic acid) for 60 minutes at 40°C at a flow rate of 300 nL/min. Eluted peptides were introduced into the nano-electrospray source at a capillary voltage of 2.0 kV. MS1 spectra were acquired for all eluted peptides in the Orbitrap mass analyzer (*R* = 120K: AGC target = 400,000; MaxIT = auto; RF Lens = 30%; mass range = 350–1500). MS/MS spectra were collected in the linear ion trap mass analyzer (rate turbo); AGC target 10,000; MaxIT = 35 ms; NCE_CID_ = 35%). The resulting MS/MS spectra were searched against a database that consists of full prion protein sequence using Mass Matrix software to identify specific sites of modification. In particular, MS/MS spectra were searched for peptides generated by pepsin digestion using mass accuracy values of 10 ppm and 0.8 Daltons for MS1 and MS/MS scans respectively, with allowed variable modifications for all known oxidative modifications previously documented for amino acid side chains [53, 54]. In addition, MS/MS spectra for each site of proposed modification were manually validated.

### Hydroxylation rate analysis

For the residue level analysis, the integrated peak areas of the unmodified peptide (*A*_u_), and each specific modified product (*A*_m_) for this peptide derived from selected ion chromatograms, were used to calculate percent modification and fraction unmodified (*F*_u_) for specific site of modification using the following equation:

$$Percent\;modification=\left[A_{m}/\left(A_{u}+\sum A_{m}\right)\right]\times 100$$

$$Fraction\;unmodified,\;F_{u}=1‐\left[A_{m}/\left(A_{u}+\sum A_{m}\right)\right]$$

Where, ∑*A*_m_ is the sum of all modified peak area from a particular peptide. The dose-response curve for each specific modification was generated using fraction unmodified for each specific site plotted versus X-ray exposure time. The modification rate constant for each specific modification was calculated by fitting the fraction unmodified for this modification site to the equation: *F*_u_(*t*) = *F*_u_(0)e^-*kt*^, where *F*_u_(0) and *F*_u_(*t*) are the fraction of unmodified residue at time zero and time t, respectively; and *k* is first order rate constant [49, 50]. The half-life (*t*_1/2_) for modification of amino acid residue was calculated using the equation: *t*_1/2_ (sec) = ln2/*k*, where *k* (sec^-1^) is the rate constant [51, 52]. For comparing the difference in exposure of the amino acid residues between recPrP and PrP^Sc^ we calculated the modification rate ratio (protection factor) by dividing the modification rate of residue in recPrP with the modification rate of same residue in PrP^Sc^ [55, 56].

### Statistical analysis

Statistical analysis was performed using SPSS Statistics 27 package (IBM) or KaliedaGraph (Synergy) software, and 3D modelling with PyMOL software. Experimental results were expressed as mean ± SEM, and a post hoc test (Dunnet) was used to calculate the P values. Significance level (α) was set to p<0.05.

## Results

### Molecular characteristics of CJD MM1, MM2, and VV2 prions

From the collection of samples obtained from 343 patients with an unequivocal diagnosis of Type 1 (MM1), Type 2 (MM2), and Type 2 (VV2) sCJD, we selected one case that is representative of each neuropathology group (**Fig 1**) and displayed ≥99% pure Type 1 or Type 2 proteinase K-resistant PrP^Sc^ (rPrP^Sc^), as detected by Western blots [28–30]. The disease duration in these representative cases, as well as biochemical characteristics of brain PrP^Sc^ associated with them (levels of total PrP^Sc^ and rPrP^Sc^, conformational stability of PrP^Sc^) correspond to the values reported previously for each group [12, 22, 29, 30] (**Table 1**).

Western blot patterns and silver staining of purified protease resistant cores (rPrP^Sc^) of MM1, MM2, and VV2 in the final fraction 8 (F8) demonstrated expected [22, 57] characteristics, the mass of unglycosylated fragments was ~21 kDa in Type 1 and ~19 kDa in Type 2 rPrP^Sc^, and Type 2 rPrP^Sc^ was not detectable with mAb 12B2 due to the missing N-terminal epitope (**Fig 1B and 1C**). The complex band patterns are due to the presence of 2, 1, or no N-linked glycosylation chains, and ~13–15 kDa C-terminal fragments (CTFs) that were more abundant in Type 2 rPrP^Sc^ and detectable in Type 1 after longer exposure (**Fig 1C**) [22]. The isolated rPrP^Sc^ was ~90% pure (**Fig 1C**) [22]. These patterns were highly reproducible upon purification of rPrP^Sc^ from different cortical areas of the same brain [22].

### Monitoring epitope modification by synchrotron generated •OHs with direct conformation-dependent immunoassay (dCDI)

Testing the existence of phenotype-specific conformational differences between human brain-derived prion strains in polymorphic fibrillar, oligomeric, and monomeric structures has been difficult due to the lack of adequate experimental approaches suitable for all these major forms. The recently developed ultrafast synchrotron •OH footprinting is suitable for structural studies of all forms [49, 50, 54, 58] and here we expanded this methodology by monitoring the rate of epitope modification with a panel of Europium labeled monoclonal antibodies in direct conformation-dependent immunoassay (dCDI) [23, 24, 26, 29, 30, 39, 59, 60]. In this protocol, the amino acid side chains modification by OH radicals generated by radiolysis of water in a synchrotron beam led to a time-dependent loss of monoclonal antibody affinity, and the rate of residue modification is proportional to extent of solvent exposure in the native prion structure.

We selected and labeled with Europium six monoclonal antibodies with linear epitopes covering major domains of the CJD prion cores. All demonstrated a parallel response to concentration of monomeric recombinant human prion protein (recPrP) in direct CDI and pg/ml sensitivity **(Fig 2A)**. The α-helical monomers of rPrP lost 95–99.9% of antibody affinity after 10ms of irradiation and the antibody signal continued dropping at a slower rate to 50ms where it reached a plateau. The phases are strikingly distinct for all six antibodies, and the least reactive after 10 ms (eg. 5B9, 3F4, 6D11), continue to be more resistant to further interference as a function of modification compared to the other 3 epitopes (8H4, MAR1, and 1E5) (**Fig 2B**). Based on our understanding of •OH chemistry, where sulfur containing residues like M and aromatics like W are quite susceptible, followed by other aromatics and aliphatic residues, the faster interference seen for some of the epitopes is quite understandable and the multiple phases are representative of the reactivity of different residue classes [61, 62]. For example, 1E5 (145–149), the fastest, has a W and a Y, in addition it is a very small epitope presumable easily disturbed. The 8H4 epitope (145–185) encompasses 1E5, the additional structural elements (150–185) presumably make the epitope more resistant to interference. The •OH modification rate of MAR1 epitope is also in the faster category with two M and C residues (**Fig 2B**).

In comparison to the α-helical monomer of recPrP, all epitopes of brain-derived sCJD MM1, MM2, and VV2 prions demonstrated several orders of magnitude slower modification rate by •OH radicals, with major differences between sCJD MM1, MM2, and VV2 prions (**Fig 2C, 2D, 2E, 2F, 2G and 2H**). With the exception of the 145–185 sequence, all domains of sCJD MM1 prions were more protected, followed by MM2 and VV2 prions. The half-life of each epitope during synchrotron irradiation calculated from exponential decay models revealed the distinctly different patterns of MM1, MM2, and VV2 prions (**Fig 2I**). We observed the highest protection of MM1 prions in two distant epitopes—aa108-112 and aa157-167, opposite trends in VV2 prions, and intermediate protection in all domains of MM2 prions.

### Replication potency of synchrotron-irradiated prions

**Fig 3:**
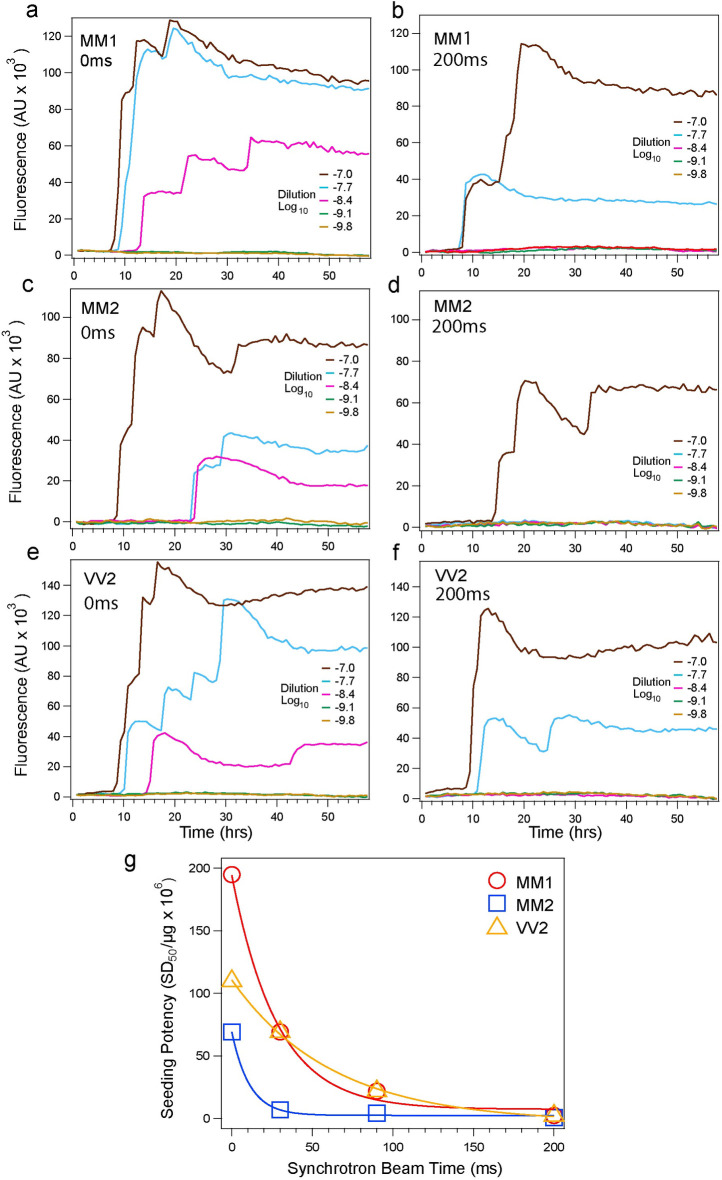
Inactivation rate of human sCJD MM1, MM2, and VV2 sCJD prions by synchrotron •OH. The end-point seeding activity of serially diluted MM1 prions (**a**) before and (**b**) after synchrotron irradiation, end-point seeding activity of serially diluted MM2 prions (**c**) before and (**d**) after irradiation, and seeding activity of serially diluted VV2 prions (**e**) before and (**f**) after synchrotron irradiation. The sCJD prions were irradiated in synchrotron for 0 to 200 ms, serially diluted, and seeding potency of each dilution was monitored by second generation RT-QuIC [41]. The curves in **a-f** are thioflavin T (ThT) fluorescence averaged at each sample dilution from four wells of 96-well RT QuIC plate as described [46]s. (**g**) Kinetics of inactivation of MM1 (red), MM2 (blue) and VV2 (yellow) sCJD prions determined as a function of exposure time. The SD_50_ was calculated with Spearman-Kärber analysis of end-point dilutions at each synchrotron irradiation time point with RT QuIC as described [46].

Evidence accumulated with prions and more recently with a growing list of other proteins indicate that the misfolded conformers can be amplified with recombinant substrates in a seeded reaction *in vitro—*a prerequisite step for misfolding propagation in the brain [22, 24, 29, 41, 46, 63–65]. At initial 10^−6^ dilution of purified prions, we observed lower ThT fluorescence, and in MM2 prions an extended lag phase after 200ms irradiation (**Fig 3**). Next, we performed systematic endpoint titration of seeding potency of synchrotron irradiated prions using second generation RT QuIC of serially diluted samples [41] (**S1 Table**). To estimate a seeding dose or dilution at which 50% of the wells became ThT positive (Log_10_ SD_50_) [46], we employed the Spearman-Kärber analysis, analogous to calculations of 50% prion lethal dose (LD_50_) in prion bioassay’s [36, 42–45]. The end-point titration data revealed a progressive drop in prion seeding activity and distinctly different seeding inactivation kinetics, with 79-, 89-, and 45-fold less seeding activity of MM1, MM2, and VV2 prions, respectively, after 200ms of irradiation. The major drop in sCJD MM1 prions seeding activity (**Fig 3**) correlates with rapid modifications of solvent-exposed amino acid side chains in 93–109, 145–185, and 179–214 domains, while the rest of the sequence is highly protected (**Fig 2**), and the MM2 prions shows similar trends. The irradiation of VV2 prions demonstrated a delayed and less significant impact on seeding activity (**Figs 2 and 3**). Cumulatively, the distinct structural organization of each sCJD prion strain appears to determine different inactivation kinetics by •OH radicals, and the rapid kinetics implicate modifications of distinctly solvent-exposed domains.

### Synchrotron •OH footprinting monitored by mass spectrometry

The dCDI monitoring of the hydroxylation rate of a given antibody epitope in human prion is a new addition to the •OH footprinting techniques and it is a function of (i) solvent accessibility in a native conformation, and (ii) different hydroxylation rates of different amino acids within the epitope sequence [61]. These attributes allow us to compare the hydroxylation rates of the same epitope in different human prions with high sensitivity, even though the hydroxylation kinetics of different epitopes is primarily driven by epitope span, sequence, and sensitivity of particular antibody to the amino acid side chain modifications. As a result, the epitopes differ in hydroxylation kinetics in the same protein and up to 100-fold in the residual signals, as demonstrated with recombinant alpha-helical monomers of PrP (**Fig 2B**).

Although dCDI monitoring of synchrotron-induced hydroxylation demonstrated high sensitivity and differential power for different prion strains, it measures the cumulative changes originating from the different residues at the domain level dictated by the sequence of antibody epitope and antibody affinity and these aspects do not allow direct comparison with inactivation rate. To determine the hydroxylation more accurately and correlate the data with inactivation at the amino acid level, the aliquots of synchrotron-irradiated sCJD MM1, MM2 and VV2 prions were, after formic acid denaturation, digested with pepsin and analyzed by MS/MS spectrometer coupled with UPLC. The optimized pepsin digestion generated overlapping peptide coverage of the prion core (**S2 Table**), and the modification rate was calculated from integrated peak areas of modified and unmodified peptides.

**Fig 4:**
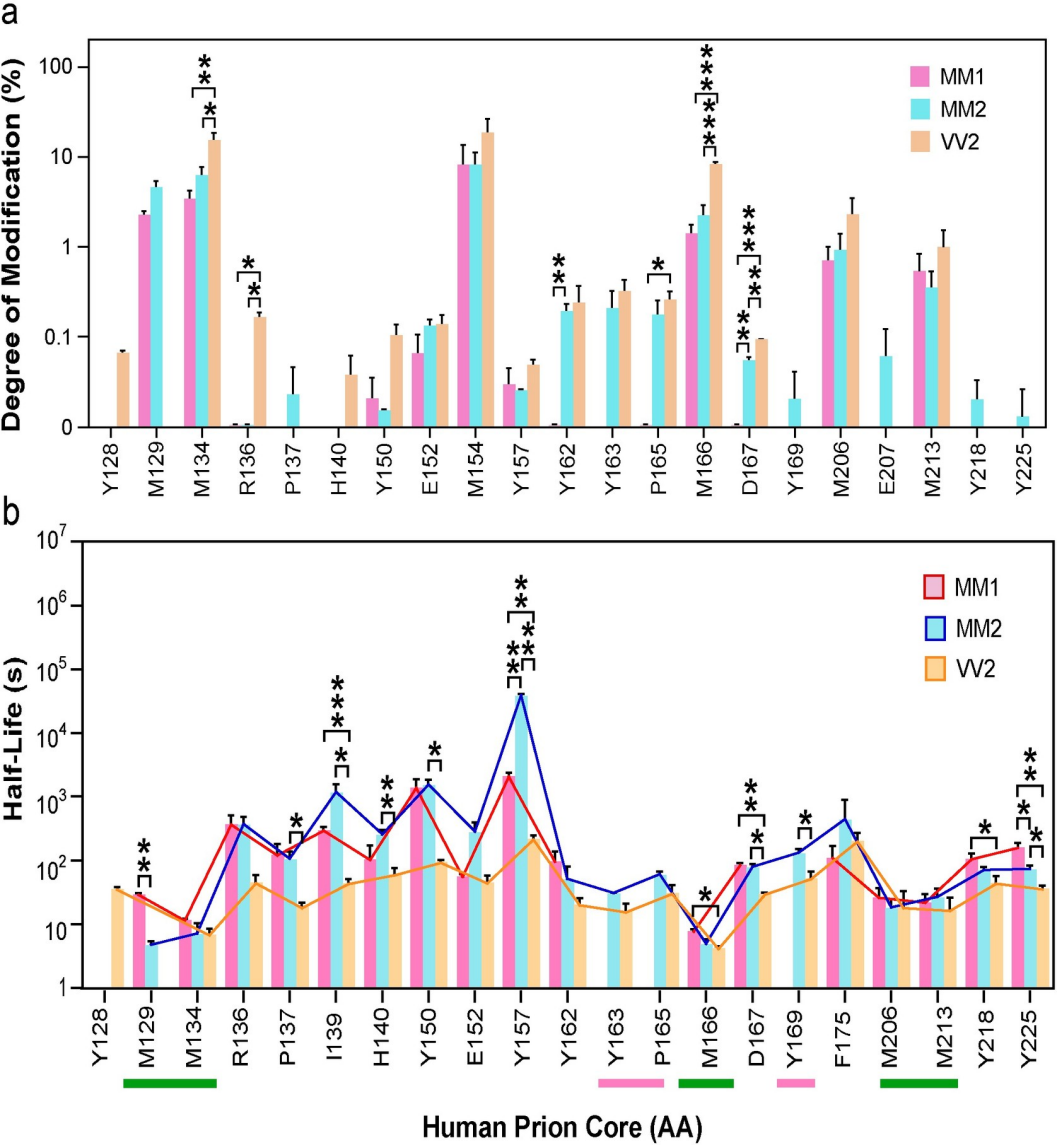
Differential •OH modification of amino acid side chains in MM1 (red), MM2 (blue) and VV2 (yellow) brain-derived sCJD prions (**a**) before and (**b**) after synchrotron irradiation. (**a**) Degree of modification of specific residues before synchrotron irradiation was calculated as described for synchrotron irradiated samples. (**b**) •OH modification half-life of specific amino acid side chains in MM1 (red), MM2 (blue) and VV2 (yellow) calculated from fitting the fraction of unmodified data using first order rate constant equation. The complete protection of residues in MM1 prions is indicated by red line; the green line indicates high modifications rates due to the solvent exposure. The values are expressed as mean ± SEM from two independent irradiation experiments and three MS/MS peptide datasets for each time point. *, p < 0.05; **, p < 0.01; and ***, p < 0.001.

The initial analysis of non-irradiated MS data revealed that some modifications of side chains, notably methionine, occurred before irradiation. The degree of hydroxylation follows the susceptibility of individual amino acids expected from the peptide experiments: Cys > Met > Trp > Tyr > Phe > Cystine > His > Leu ~ Ile > Arg ~ Lys ~ Val > Ser ~ Thr ~ Pro > Gln ~ Glu > Asp ~ Asn > Ala > Gly [61, 66]. High relative reactivity of methionine and high content in human prion core (10 out of 137 amino acids is methionine), this amino acid is a particularly useful indicator of solvent accessibility [35, 61, 66, 67] (**Fig 4A and S2 and S3 Tables**). The sCJD VV2 prions have the highest proportion of hydroxylated residues followed by MM2 and MM1, even though the purification was performed at low temperatures and buffers that do not generate •OHs. Cumulatively, the modification of amino acids 129–136, M154, and 162–167 residues in MM1, MM2, and VV2 prions indicate that these domains were exposed to •OHs during the accumulation in human brains in vivo. Whether this effect is due to the extended half-life of different prions in the brain we observed in rodent models [68], disease-induced oxidative stress, or combination of both will require time course study in transgenic mice.

The residue specific modification rates of MM1, MM2 and VV2 calculated by plotting exposure time vs fraction of unmodified peptides using the first order rate equation were determined for 23 residues (**S3 Table)** spanning the prion core sequence downstream from residue Y128 (**Figs 4B and** S1). Unfortunately, ragged cleavage sites generated at the N-terminus by Proteinase K during purification [69] did not yield reproducible peptide coverage upstream from Y128 residue (**Figs 1 and 2**). The sCJD MM1, MM2, and VV2 prion strains showed significant differences in oxidation rate profiles indicative of variable exposure to the solvent (**Figs 4B and** S1). As shown previously with dCDI, the VV2 prions demonstrated the highest rates of hydroxylation through the whole core, followed by MM2, and MM1 prions, indicating that the VV2 prions have the least protected amino acid side chains in a unique structural organization. The hydroxylation expressed as rates (**S1 Fig and S3 Table**) and half-life (**Fig 4B**) varied broadly within 4 orders of magnitude and highly protected residues clustered within three distinct domains: amino acids 136–162, 167–175, and 218–225. In contrast to MM2 and VV prions, the Y163, P165, and Y169 residues were completely protected in MM1 prions and thus the half-life couldn’t be calculated (**Figs 4B and** S1). There is no difference in hydroxylation rates of residues M134, Y162, M206, M213 and Y218 in MM1, MM2 and VV2 prions, which suggest that these residues are forming a structural motif common to all three human prion strains (**Fig 4B**). The rapid rate of hydroxylation in domains 129–134, M166, and 206–213 correlated with rapid inactivation of MM1 and MM2 prions in seeding RT QuIC assays (**Fig 3D**). Notably, those are the residues that underwent hydroxylation before synchrotron irradiation, and implicate differential modification rates during the accumulation in the brains (**Fig 4A**).

**Fig 5:**
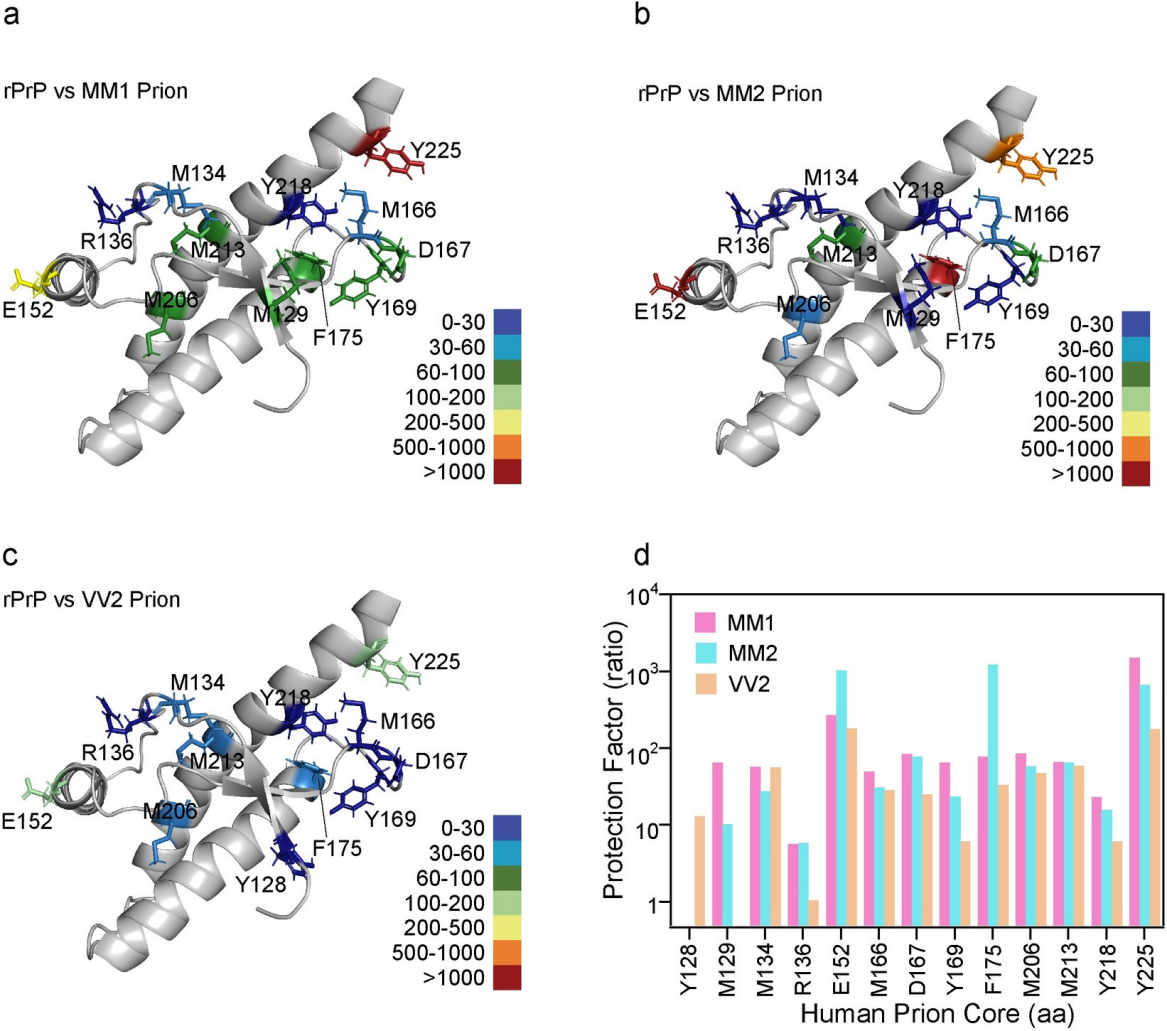
Relative protection factor of MM1, MM2, and VV2 sCJD prions. The protection factor for individual residues was calculated from the modification rate of •OH modification of amino acid side chains in α-helical monomeric recHuPrP divided by the modification rate of the same residues in (**a**) MM1, (**b**) MM2, and (**c**) VV2 prions; the different range of amino acid protection is indicated by the color scale. (**d**) Comparison of protection factors in three strains of sCJD prions.

Next, we calculated the protection factor by dividing the modification rate of residue in recombinant human prion protein (recPrP) containing either 129M or 129V (**S1 Fig** and **S4 Table)** by modification rate of same residue in PrP^Sc^ (MM1, MM2 and VV2) and model the residue distribution using the solution NMR structure of human recPrP (1QM0) deposited in PDB [70]. The model is indicative of a major alteration in solvent accessibility of surface residues during conversion of monomeric prion protein where the helix structures of the monomer become buried in presumably beta sheet structured prion particles (**Fig 5A, 5B, 5C and 5D**). With the exception of R136 in VV2 prions, all other monitored residues underwent a major strain-specific structural rearrangement in the conformational transition from α-helical monomer to beta-sheet structured prion particles, resulting in a high protection from solvent. The amino acid side chains in a 166–175 domain of human prions are relatively exposed to the solvent, and the residue F175 shows a major difference in the accessibility in MM1, MM2, and VV2 strains of human prions. Notably, the 165–177 domain is forming a beta2-alfa2 loop in the cellular prion protein (PrPC) and dramatically influences the interspecies prion transmission of sheep scrapie, BSE, mouse-adapted scrapie, deer chronic wasting disease, and hamster-adapted scrapie prions [71]. This loop may exist in two alternative conformations (3_10_-helix turn or type I beta-turn) and for example replacement of Y169G result in a PrPC containing only the type—I beta-turn loop—structure [71, 72] and different interspecies transmission profile of prions. Cumulatively, these and our data suggest that this particular domain plays a major role in the conformational transition from cellular prion protein to prions, and thus determine the infectivity and interspecies transmissibility of human prions as well.

## Discussion

The misfolded human prion protein, PrP^Sc^, is the essential, and possibly sole component of infectious human prions [24]. However, a high-resolution structure of human prions is lacking, and the key molecular mechanisms that determine initiation and propagation under physiological conditions—human prion infectivity—remain obscure [22, 24, 73]. Our investigations of the structure and replication potency of major human prions with synchrotron •OH footprinting monitored with MS and dCDI in this report allowed us to establish the fundamental structural attributes of infectivity of the major human brain-derived prions. Our present study demonstrates, for the first time, that replication as well as inactivation of different strains of infectious human prions is primarily driven by the distinct structural organization of their external domains. Furthermore, the major differences in infectivity, as well as inactivation susceptibility, appear to be controlled by seemingly subtle differences in the conformation, especially in the N- terminal and C-terminal domain, which in PrP is involved in posttranslational modifications [9–11, 23].

Human prion diseases are conceivably the most heterogenous neurodegenerative disorders and a growing body of research indicates that they are caused by distinct strains of human prions [9–11, 22]. However, the structural studies of human prions have lagged behind the recent progress in rodent laboratory prions, in part because of their complex molecular characteristics and prohibitive biosafety requirements for laboratory experiments and bioassays, due to the human-to-human transmissibility of disease which is invariably fatal and has no treatment. The early discovery of distinct polymorphic PK cleavage sites in human brain-derived PrP^Sc^ has been used for the clinicopathological diagnostic classifications of sCJD cases; and in concert with the codon 129 PRNP haplotype, the different protease-resistant fragments of PrP^Sc^ protein (rPrP^Sc^) types broadly correlate with distinct disease phenotypes (**Fig 1**) [74–79]. The majority of sCJD patients are homozygous for methionine in codon 129 of the PRNP gene, accumulate Type 1 rPrP^Sc^, with disease duration of approximately 4 months (**Fig 1**) and fine punctate (synaptic) deposits of PrP^Sc^ [36, 75]. In contrast, patients with the second most frequent phenotype are homozygous for valine in codon 129 of the PRNP gene, accumulate Type 2 PrP^Sc^ and manifest a different disease course, with early ataxia, predominant extrapyramidal symptoms, relatively late-onset dementia in the extended course of the disease, and large plaque-like deposits of PrP^Sc^ [11, 75]. The sCJD VV2 subtype has typically the longer duration, with major deposits of plaque forming PrP^Sc^ in the cerebellum [9–11].

The molecular mechanisms that are being debated in support of human prion strain phenomenon are (i) packing polymorphism, in which the same segment of protein guides alternative packing of β-sheets [80, 81]; (ii) segmental polymorphism, in which a different segment of protein encodes different β-sheet formation [82]; and (iii) structural and kinetic effect of different auxiliary ligands [24]. However, the high resolution structure of human prions is lacking and our recent hydrogen/deuterium amide exchange data on sCJD MM1, MM2, and synthetic human prions provide evidence that human prions differ significantly from noninfectious prion protein amyloid and rodent prions [22–24]. Furthermore, our data indicate that, in contrast to previous observations on yeast and some laboratory rodent prion strains, the replication rate of sCJD prions is primarily determined not by conformational stability (or fragmentation susceptibility), but by structural features that control the growth rate of prion protein aggregates [22, 24].

The differential, but generally very slow exchange rate of amide bonds in peptides covering 128–162, and 167–214 domains is indicative that these two domains are forming a b-sheet structure in human prions; this supports the in-register parallel model observed with solid state NMR in a recombinant C-terminally truncated fragment of mouse PrP23-144 [23, 83]. The early radiobiological investigations on the resistance of prions to inactivation by ionizing radiation, played a critical role in establishing the small target size [84, 85], and the independence of infectivity on nucleic acid [86], and thus these findings provided a foundation for the formulation of the prion concept as a self-replicating protein [1]. Building on these approaches, we identified three domains— Y128-M134, Y163-M166, and M206-M213—with a high rate of ^*•*^OH modification during synchrotron radiolysis, indicating significant solvent exposure that correlated with inactivation of prion seeding activity. Prior MS studies of human brain-derived prion fragments generated with Proteinase K identified additional truncated C-terminal fragments (CTFs): 13kDa CTF fragment with cleavage site in residues 154–156, and 12kDa fragment starting at residues 162–167 of human brain-derived PrPSc [22, 87]. These and our data on solvent accessibility suggest that these residues are forming in human prions a solvent and enzyme exposed loop. Additional cleavage sites in residue 134 and 169 found in laboratory rodent prions correspond to solvent exposed domains in human prions, but the rest does not and indicate significant structural differences between human and rodent prions we observed previously with HX MS [22, 88].

All three solvent-exposed human prion domains contain, or are neighbors of, P137, P158, and P165 residues suggesting that they might be involved in forming a loop or turn into a beta sheet structure. Additionally, while comparing solvent accessibility of proline residue between MM1, MM2 and VV2, we found a significant difference in microenvironment around P137 and P165. Such differences in proline residue environment may have a pronounced effect on the tertiary as well as quaternary structure; this might be one of the driving factors that contribute to the formation of prion strains with distinctly different affinity for a substrate PrP^C^, and thus different replications rates and toxicity. These structural and inactivation data may open new potential therapeutic strategies for human prion diseases that would target the common solvent-exposed elements of human prions in an early interphase of PrP^Sc^/PrP^C^ interaction.

## Supporting information

S1 Fig Hydroxyl radical modification rate of residues in (a) α-helical monomers of recHuPrP(129M) and rPrP(129V), and (b) sCJD MM1, MM2, and VV2 prions.
(TIF)

S1 Table End point titration of human prion seeding activity after synchrotron irradiation with RT QuIC. Samples were diluted five-fold and the seeding activity of each sample was monitored in four wells for 60 hrs.
(DOCX)

S2 Table Fragments of MM1, MM2 and VV2 sCJD prions and their sequences showing variable degree of modification rate. Residues colored with green, blue, red and black represents modification rate as >0.0375, 0.0375–0.0075, 0.0075–0.00015 and <0.00015 respectively.
(DOCX)

S3 Table Hydroxyl radical modification rates of MM1, MM2 and VV2 sCJD prions. Note codon 129 polymorphism in 128–133 peptide.
(DOCX)

S4 Table Hydroxyl radical modification rate of residues in monomeric a-helical recHuPrP^C^(129M) and rPrP^C^(129V). (DOCX)
