# Supplementary figures and images for: Structurally distinct external solvent-exposed domains drive replication of major human prions
Source: PLoS Pathog. 2021 Jun 17;17(6):e1009642. doi: 10.1371/journal.ppat.1009642 (PMC8211289; doi:10.1371/journal.ppat.1009642)

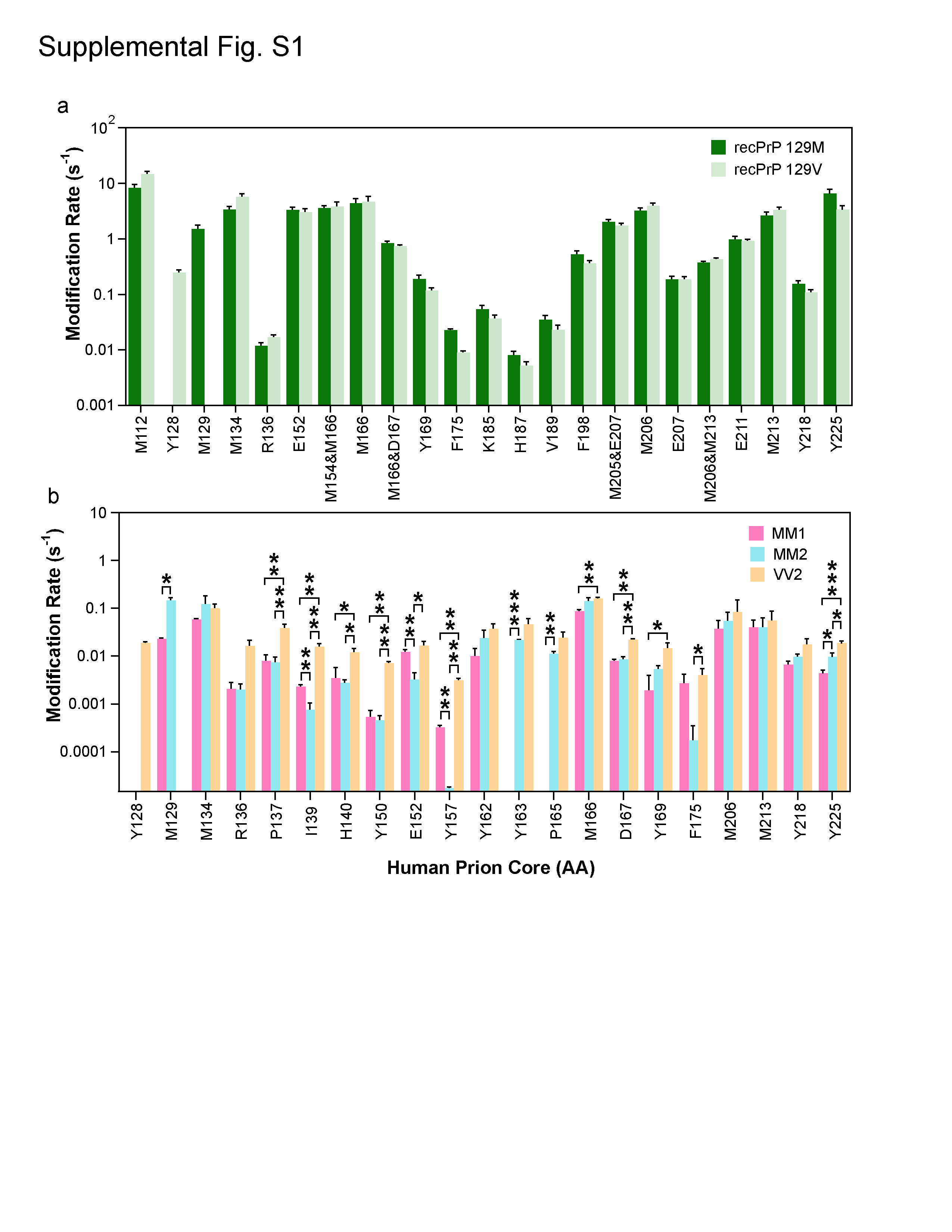

Supplement: S1 Fig — Hydroxyl radical modification rate of residues in (a) α-helical monomers of recHuPrP(129M) and rPrP(129V), and (b) sCJD MM1, MM2, and VV2 prions. (TIF) [file ppat.1009642.s001.tif]
