# Supplementary material for: Structurally distinct external solvent-exposed domains drive replication of major human prions
Source: PLoS Pathog. 2021 Jun 17;17(6):e1009642. doi: 10.1371/journal.ppat.1009642 (PMC8211289; doi:10.1371/journal.ppat.1009642)
Supplement: S1 Table — Samples were diluted five-fold and the seeding activity of each sample was monitored in four wells for 60 hrs. (DOCX) [file ppat.1009642.s002.docx]

**S1 Table**. End point titration of human prion seeding activity after synchrotron irradiation with RT QuIC. Samples were diluted five-fold and the seeding activity of each sample was monitored in four wells for 60 hrs.

| **Sample** | **Dilution** | **MM1** | | **MM2** | | **VV2** | |
| --- | --- | --- | --- | --- | --- | --- | --- |
|  | **Log_10_** | **Positive Wells** | **Avg. Max ThT Fluorescence** | **Positive Wells** | **Avg. Max ThT Fluorescence** | **Positive Wells** | **Avg. Max ThT Fluorescence** |
| MM1 Positive Control | -7.7 | 4/4 | 179636 | 4/4 | 228857 | 4/4 | 228638 |
| No Seed | - | 0/4 | 457 | 0/4 | 1077 | 0/4 | 1431 |
| Synchrotron Beam 0ms | -7.0 | 4/4 | 125585 | 4/4 | 111107 | 4/4 | 151765 |
|  | -7.7 | 4/4 | 120961 | 2/4 | 43787 | 4/4 | 129060 |
|  | -8.4 | 3/4 | 62325 | 1/4 | 31229 | 2/4 | 38673 |
|  | -9.1 | 0/4 | 375 | 0/4 | 912 | 0/4 | 1485 |
|  | -9.8 | 0/4 | 210 | 1/4 | 30112 | 0/4 | 2240 |
| 30ms | -7.0 | 4/4 | 124808 | 2/4 | 52324 | 4/4 | 172742 |
|  | -7.7 | 4/4 | 118579 | 1/4 | 36969 | 3/4 | 105383 |
|  | -8.4 | 2/4 | 75239 | 1/4 | 27446 | 1/4 | 26470 |
|  | -9.1 | 2/4 | 28913 | 0/4 | 1972 | 0/4 | 2046 |
|  | -9.8 | 1/4 | 19239 | 0/4 | 2030 | 0/4 | 3210 |
| 90ms | -7.0 | 4/4 | 125428 | 3/4 | 56202 | 4/4 | 120540 |
|  | -7.7 | 4/4 | 136619 | 0/4 | 450 | 2/4 | 94730 |
|  | -8.4 | 1/4 | 21904 | 3/4 | 79106 | 0/4 | 1456 |
|  | -9.1 | 0/4 | 495 | 0/4 | 2318 | 0/4 | 1745 |
|  | -9.8 | 0/4 | 1053 | 0/4 | 2668 | 0/4 | 2573 |
| 200ms | -7.0 | 4/4 | 112730 | 3/4 | 68708 | 3/4 | 121432 |
|  | -7.7 | 1/4 | 41076 | 0/4 | 1895 | 2/4 | 52592 |
|  | -8.4 | 0/4 | 3387 | 0/4 | 1327 | 0/4 | 1359 |
|  | -9.1 | 1/4 | 25994 | 0/4 | 2848 | 0/4 | 2794 |
|  | -9.8 | 0/4 | 3538 | 0/4 | 1965 | 0/4 | 2201 |
