# Supplementary material for: Structurally distinct external solvent-exposed domains drive replication of major human prions
Source: PLoS Pathog. 2021 Jun 17;17(6):e1009642. doi: 10.1371/journal.ppat.1009642 (PMC8211289; doi:10.1371/journal.ppat.1009642)
Supplement: S2 Table — Residues colored with green, blue, red and black represents modification rate as >0.0375, 0.0375–0.0075, 0.0075–0.00015 and <0.00015 respectively. (DOCX) [file ppat.1009642.s003.docx]

| **Peptides** | **Peptic fragments** | | |
| --- | --- | --- | --- |
|  | **MM1** | **MM2** | **VV2** |
| **128-133** | - | - | **Y**VLGSA |
| **128-133** | Y**M**LGSA | Y**M**LGSA | - |
| **132-144** | SA**M**SRPIIHFGSD | SA**M**SRPIIHFGSD | SA**M**SRPIIHFGSD |
| **133-144** | AMS**RP**IIHFGSD | AMS**RP**IIHFGSD | AMS**RP**IIHFGSD |
| **134-144** | MSR**PIIH**FGSD | MSR**PIIH**FGSD | MSR**PIIH**FGSD |
| **135-144** | S**R**PIIHFGSD | S**R**PIIHFGSD | S**R**PIIHFGSD |
| **150-160** | **Y**R**E**N**M**HR**Y**PNQ | **Y**R**E**N**M**HRYPNQ | **Y**R**E**N**M**HR**Y**PNQ |
| **161-168** | V**Y**YRP**MD**E | V**Y**YR**PMD**E | V**Y**YR**PMD**E |
| **162-168** | YYRP**M**DE | Y**Y**R**PM**DE | Y**Y**R**PM**DE |
| **169-178** | YSNQNN**F**VHD | **Y**SNQNN**F**VHD | **Y**SNQNN**F**VHD |
| **169-181** | **Y**SNQNNFVHDCVN | **Y**SNQNNFVHDCVN | **Y**SNQNNFVHDCVN |
| **206-213** | **M**ERVVEQ**M** | **M**ERVVEQ**M** | **M**ERVVEQ**M** |
| **218-225** | **Y**ERESQA**Y** | **Y**ERESQA**Y** | **Y**ERESQA**Y** |

**S2 Table.** Fragments of MM1, MM2 and VV2 sCJD prions and their sequences showing variable degree of modification rate. Residues colored with green, blue, red and black represents modification rate as >0.0375, 0.0375-0.0075, 0.0075-0.00015 and <0.00015 respectively.
