# Supplementary material for: Structurally distinct external solvent-exposed domains drive replication of major human prions
Source: PLoS Pathog. 2021 Jun 17;17(6):e1009642. doi: 10.1371/journal.ppat.1009642 (PMC8211289; doi:10.1371/journal.ppat.1009642)
Supplement: S3 Table — Note codon 129 polymorphism in 128–133 peptide. (DOCX) [file ppat.1009642.s004.docx]

**S3 Table.** Hydroxyl radical modification rates of MM1, MM2 and VV2 sCJD prions. Note codon 129 polymorphism in 128-133 peptide.

| **Peptides** | **Sequence** | **Oxidized Residues** | **MM1  (s^-1^)** | **MM2  (s^-1^)** | **VV2 (s^-1^)** |
| --- | --- | --- | --- | --- | --- |
|  |  |  |  |  |  |
| **128-133** | **Y**VLGSA | Y128 | - | - | 0.019 ± 0.0011 |
| **128-133** | Y**M**LGSA | M129 | 0.0231 ± 0.0008 | 0.1475 ± 0.0195 | - |
| **132-144** | SA**M**SRPIIHFGSD | M134 | 0.0589 ± 0.0024 | 0.124 ± 0.0572 | 0.102 ± 0.0202 |
| **133-144** | AMS**RP**IIHFGSD | R136_P137 | 0.0023 ± 0.0023 | 0.0276 ± 0.00085 | 0.0302 ± 0.00385 |
| **134-144** | MSR**PIIH**FGSD | I138_I139 | 0.00232 ± 0.000229 | 0.000761 ± 0.000299 | 0.016 ± 0.00204 |
|  |  | H140 | 0.00357 ± 0.00222 | 0.00281 ± 0.000416 | 0.0122 ± 0.00226 |
|  |  | P137 | 0.00798 ± 0.00256 | 0.00757 ± 0.00198 | 0.0394 ± 0.00667 |
| **135-144** | S**R**PIIHFGSD | R136 | 0.0021 ± 0.000735 | 0.00203 ± 0.000585 | 0.0165 ± 0.0048 |
| **150-160** | **Y**R**E**N**M**HR**Y**PNQ | E152 | 0.0124 ± 0.0012 | 0.00328 ± 0.00147 | 0.0168 ± 0.00344 |
|  |  | M154_Y157 | 0.000327 ± 0.0000325 | 0.0000177 ± 0.00000079 | 0.00313 ± 0.000335 |
|  |  | M154_Y150 | 0.000549 ± 0.000181 | 0.000467 ± 0.000098 | 0.00727 ± 0.000405 |
| **161-168** | V**Y**YR**PMD**E | P165 | 0 | 0.0157 ± 0.0004 | 0.0124 ± 0.0018 |
|  |  | Y162 | 0.0102 ± 0.00432 | 0.0241 ± 0.0108 | 0.0378 ± 0.00954 |
|  |  | M166_D167 | 0.00805 ± 0.00047 | 0.00872 ± 0.00088 | 0.0226 ± 0.000399 |
| **162-168** | Y**Y**R**PM**DE | M166 | 0.0883 ± 0.0053 | 0.144 ± 0.0225 | 0.163 ± 0.007 |
|  |  | P165 | 0 | 0.0114 ± 0.00105 | 0.0245 ± 0.00765 |
|  |  | Y163 | 0 | 0.0223 ± 0.0000999 | 0.0472 ± 0.0145 |
| **169-178** | **Y**SNQNN**F**VHD | Y169 | 0 | 0.0042 ± 0.00134 | 0.0248 ± 0.00536 |
|  |  | F175 | 0.002747 ± 0.00138 | 0.000174 ± 0.000174 | 0.00411 ± 0.00141 |
| **169-181** | **Y**SNQNNFVHDCVN | Y169 | 0.00196 ± 0.00196 | 0.00547 ± 0.000919 | 0.0148 ± 0.00406 |
| **206-213** | **M**ERVVEQ**M** | M213 | 0.0404 ± 0.0166 | 0.041 ± 0.0217 | 0.0564 ± 0.0299 |
|  |  | M206 | 0.0379 ± 0.018 | 0.0555 ± 0.0268 | 0.0846 ± 0.0635 |
| **218-225** | **Y**ERESQA**Y** | Y218 | 0.00673 ± 0.00104 | 0.00987 ± 0.00106 | 0.0178 ± 0.00545 |
|  |  | Y225 | 0.00445 ± 0.000633 | 0.00989 ± 0.00159 | 0.019 ± 0.00213 |
