# Supplementary material for: Structurally distinct external solvent-exposed domains drive replication of major human prions
Source: PLoS Pathog. 2021 Jun 17;17(6):e1009642. doi: 10.1371/journal.ppat.1009642 (PMC8211289; doi:10.1371/journal.ppat.1009642)
Supplement: S4 Table — (DOCX) [file ppat.1009642.s005.docx]

| **Peptides** | **Sequence** | **Oxidized Residues** | **rPrP^C^ - 129M (s^-1^)** | **rPrP^C^ - 129V (s^-1^)** |
| --- | --- | --- | --- | --- |
|  |  |  |  |  |
| **112-127** | **M**AGAAAAGAVVGGLGG | M112 | 8.38 ± 1.1 | 14.82 ± 1.59 |
| **128-133** | Y**M**LGSA | M129 | 1.52 ± 0.24 | - |
|  | **Y**VLGSA | Y128 | - | 0.25 ± 0.027 |
| **132-144** | SA**M**SRPIIHFGSD | M134 | 3.41 ± 0.44 | 5.81 ± 0.68 |
| **134-144** | **M**S**R**PIIHFGSD | M134 | 3.15 ± 0.32 | 4.05 ± 0.67 |
|  |  | R136 | 0.012 ± 0.00146 | 0.0173 ± 0.00145 |
| **150-168** | YR**E**N**M**HRYPNQVYYRP**M**DE | E152 | 3.38 ± 0.31 | 3.06 ± 0.44 |
|  |  | M154&M166 | 3.64 ± 0.3 | 3.84 ± 0.77 |
| **156-168** | RYPNQVYYRP**M**DE | M166 | 4.44 ± 0.89 | 4.7 ± 1.12 |
|  |  | M166&D167 | 0.847 ± 0.0553 | 0.745 ± 0.0364 |
| **161-168** | VYYRP**MD**E | M166&D167 | 0.686 ± 0.0472 | 0.564 ± 0.0235 |
| **169-178** | **Y**SNQNN**F**VHD | Y169 | 0.193 ± 0.0281 | 0.118 ± 0.0128 |
|  |  | F175 | 0.023 ± 0.0009 | 0.00907 ± 0.00055 |
| **169-181** | **Y**SNQNN**F**VHDCVN | Y169 | 0.129 ± 0.017 | 0.0924 ± 0.012 |
|  |  | F175 | 0.214 ± 0.0012 | 0.138 ± 0.0018 |
| **182-197** | ITI**K**Q**H**T**V**TTTTKGEN | K185 | 0.055 ± 0.0085 | 0.0376 ± 0.00491 |
|  |  | H187 | 0.00813 ± 0.00129 | 0.00532 ± 0.000822 |
|  |  | V189 | 0.0353 ± 0.00662 | 0.0233 ± 0.00447 |
| **182-202** | ITIKQHTVTTTTKGEN**F**TETD | F198 | 0.536 ± 0.0766 | 0.368 ± 0.0406 |
| **198-207** | FTETDVK**MME** | M206 | 3.25 ± 0.33 | 4.02 ± 0.39 |
|  |  | M205&E207 | 2.05 ± 0.198 | 1.76 ± 0.14 |
|  |  | M205&M206&E207 | 0.616 ± 0.0248 | 0.598 ± 0.0251 |
| **206-213** | **ME**RVV**E**Q**M** | M213 | 1.6 ± 0.12 | 1.33 ± 0.092 |
|  |  | E211 | 0.99 ± 0.12 | 0.93 ± 0.055 |
|  |  | E207 | 0.19 ± 0.022 | 0.19 ± 0.02 |
| **206-214** | **M**ERVVEQ**M**C | M213 | 2.7 ± 0.32 | 3.37 ± 0.32 |
|  |  | M206_M213 | 0.38 ± 0.014 | 0.44 ± 0.013 |
| **206-217** | **M**ERVVEQ**M**CITQ | M213 | 17.41 ± 2.3 | 26.42 ± 5.63 |
|  |  | M206_M213 | 2.75 ± 0.25 | 3.15 ± 0.24 |
| **215-224** | ITQ**Y**ERESQA | Y218 | 0.157 ± 0.0192 | 0.11 ± 0.0119 |
| **218-231** | YERESQA**Y**YQRGSS | Y225 | 6.71 ± 1.13 | 3.44 ± 0.53 |

**S4 Table.** Hydroxyl radical modification rate of residues in monomeric a-helical recHuPrP^C^(129M) and rPrP^C^(129V).

“-” indicates no corresponding fragment with residue “Valine” in rPrP^C^ - 129M whereas “Methionine” in rPrP^C^ - 129V, at 129^th^ position.
